# Factors associated with pet dog behavior in Thailand

**DOI:** 10.14202/vetworld.2023.957-964

**Published:** 2023-05-09

**Authors:** Worakan Boonhoh, Tuempong Wongtawan, Prarom Sriphavatsarakom, Natalie Waran, Chaiwat Boonkaewwan

**Affiliations:** 1 College of Graduate Studies, Walailak University, Nakhon Si Thammarat, 80160, Thailand; 2 Small Animal Research Group, Akkhraratchakumari Veterinary College, Walailak University, Nakhon Si Thammarat, 80160, Thailand; 3 Center of Excellence in Innovation on Essential Oil and Bioactive Compounds, Walailak University, Nakhon Si Thammarat, 80160, Thailand; 4 One Health Research Center, Walailak University, Nakhon Si Thammarat, 80160, Thailand; 5 Department of Preclinic and Applied Animal Science, Faculty of Veterinary Science, Mahidol University, Nakhon Pathom, 73170, Thailand; 6 Faculty of Education, Humanities and Health Science, Eastern Institute of Technology, Hawke’s Bay, 4142, New Zealand

**Keywords:** canine behavioral assessment and research questionnaire, dog behavior, questionnaire, dog-human relationships, Thai dogs

## Abstract

**Background and Aim:**

Dog behavior problems pose serious public health and economic and animal welfare concerns. There are many factors influencing dog behavior. This study aims to explore factors associated with pet dog behavior in Thailand using the Canine Behavioral Assessment and Research Questionnaire (C-BARQ).

**Materials and Methods:**

The Thai version of the C-BARQ was validated in 2022. The online C-BARQ survey (Google Forms) was advertised and distributed on social media for 3 months. There were a total of 1827 respondents to the survey. The relationship among 12 subscales and variables was analyzed using Spearman’s correlation.

**Results:**

“Excitability,” “attachment- and attention-seeking,” and “chasing” were the three most reported behavior problems. “Trainability,” a favored dog behavior, also had a high score in the study. These behaviors were associated with the owner’s age, house type, the dog’s historical background, the number of dogs and the presence of other species in the household, the dog’s training, diet types, and the daily number of meals. The lowest mean score was for “owner-directed aggression,” and it was associated with the dog’s gender and size, the number of dogs and the presence of other species in the household, the dog’s training, and the number of daily meals.

**Conclusion:**

This is the first empirical study demonstrating factors related to dog behavior in Thailand. It provides an in-depth understanding of the prevalence and factors associated with Thai pet dog behavior and important knowledge for further studies to advocate for dog–human relationships and contribute to a reduction in dog abandonment in Thailand.

## Introduction

Humans have domesticated and selectively bred dogs for different appearances and purposes [1]. Companionship is the primary reason for the adoption of dogs today by humans [2, 3]. Dog behavior problems, which include unacceptable behaviors to the owners, are a serious concern to public health and economic and animal welfare [4]. Aggressive behavior and biting incidences among Thai dogs are often reported in the media, and these behaviors can be found among both pet and stray dogs. In the Bangkok area of Thailand, 5.3% of human injuries recorded in hospital emergency departments are attributed to dog bites [5]. Dog behavior problems can distress owners and disrupt dog–human bonds, which can lead to the contemplation of abandoning or euthanizing pet dogs [6]. Common behavior problems among dogs include aggression, destructiveness, elimination, fearfulness, and separation-related behaviors [7]. Many factors are associated with these behaviors, including the dog’s characteristics and demographic and socioeconomic factors [8].

The Canine Behavioral Assessment and Research Questionnaire (C-BARQ), which was developed and first published in English in the United States, is a common questionnaire used to evaluate dog behaviors [9]. The C-BARQ allows dog owners to evaluate their dog’s behavior in various situations and score them for frequency and severity of the behavior using a five-point Likert-type scale. Hsu and Sun [10] conducted the C-BARQ survey in Taiwan and revealed that the age and gender of dogs, the owner’s gender, types of houses, and types of residential areas were all associated with aggressive dog behavior. Dog- and stranger-directed aggression reported in northern Mexico and Brazil have been associated with intrinsic and environmental variables, such as reproductive status, dog age, and dog breed [11, 12]. Moreover, diet has also been linked to aggressive behavior [13]. Separation-related behavior, determined based on behaviors including vocalization, destructiveness, and house soiling, has been associated with the breed and age of dog and the gender of the owner [12, 14, 15]. In addition, first-time dog ownership was associated with excitement and nervousness in dogs [3]. Finally, small breeds were significantly correlated with higher excitement behaviors, such as overexcitement, biting, rushing toward or chasing people, jumping up on people, constant running around, and digging, compared to large breed dogs [3].

The Thai version of the C-BARQ was validated in 2022 by Boonhoh *et al*. [16]. This survey was shared online, and the data were collected from throughout Thailand using Google Forms (Google LLC, Mountain View, CA, USA). Approximately one-third of households in Thailand have a dog or multiple dogs [17], which the central region, including Bangkok, has the most dogs [16]. A majority of owners in Thailand are females aged 31–40 years. Both male and female pet dogs are roughly equal. The most popular dog breed in Thailand is “mixed breed,” which is categorized as medium size (10–25 kg) [16]. However, data concerning the behavioral problems of pet dogs in Thailand are scarce. This includes sociodemographic data and cultural information that may reveal associations with various pet dog behaviors.

Therefore, this study aimed to explore and reveal factors affecting pet dog behavior in Thailand. These findings will provide essential information regarding behavioral problems among dogs, can support the management of clinical behavioral problems, and serve as a foundation for relevant future studies.

## Materials and Methods

### Ethical approval and Informed consent

The study was approved by the Human Research Ethics Committee of Walailak University (WUEC-21-253-01). All respondents read and accepted the consent form through an online document at the beginning of the online questionnaires.

### Study period and location

The Thai version of the C-BARQ survey was conducted as an online survey using Google Form (Google LLC, Mountain View, CA, USA) from November 29^th^ and December 31^st^, 2021. The questionnaire was advertised both online and on-site all over Thailand. For online advertising, we used Facebook, Instagram, Line, and websites that were related to veterinarians and dogs. For onsite advertising, we posted the QR code to many animal hospitals, which allows the respondent to use their mobile phone to scan the code and answer the questionnaire.

### Respondents

The participation criteria for the study were as follows: (1) Respondents should be Thai residents with a minimum age of 18 years and (2) they must have owned a dog for more than 1 month, and the dog’s age must be at least 2 months old. Owners with multiple dogs were asked to answer separate questionnaires for each dog. The survey included sociodemographic questionnaires (Table-1) and the Thai C-BARQ [16] (Table-2). A total of 1827 responses were submitted to the survey.

**Table-1:** Sociodemographic variables.

Index	Detail	Index	Detail
Owners’ gender	1. Female 2. Male	Home types	1. Home with yard 2. Home with no yard
Dog owner age	1. 18–22 years old 2. 23–30 years old 3. 31–40 years old 4. 41–50 years old 5. 51–60 years old 6. >60 years old	Dog size	1. Toy breed 2. Small breed 3. Medium breed 4. Large breed 5. Giant breed
Dog age	1. 1–5 months old 2. 6–12 months old 3. 13–23 months old 4. 2–6 years old 5. 7–9 years old 6. >9 years old	Number of dogs in the household	1. 1 dog 2. 2 dogs 3. 3 dogs 4. 4 or more dogs
Dog gender	1. Female 2. Male	Dog reproductive status	1. Intact 2. Neutered
Dog training	1. Yes 2. No	Other species of animals in the household	1. None 2. Has other species in the household
Dog walking routine	1. None 2. 1–3 times/week 3. 4–7 times/week 4. >7 times/week	Diet types	1. Conventional diet 2. Homemade diet 3. Biological appropriate raw foods
Duration of dogs was left alone at houses per day	1. None 2. 1–3 h/day 3. 3–6 h/day 4. 6–9 h/day 5. 9–12 h/day 6. >12 h/day	Number of meal per day	1. 1 meal/day 2. 2 meals/day 3. 3 meals/day 4. ≥4 meals/day 5. *ad libitum*

**Table-2:** The formulae used to calculate 12 behavior subscale scores of Thai C-BARQ.

Subscales	Formulae
Stranger-directed aggression	Questionnaire items (10+11+12+15+16+18+20+21+22+28)/10
Unfamiliar dog/human-directed fear	Questionnaire items (36+37+39+40+45+46+52+53)/8
Owner-directed aggression	Questionnaire items (13+14+17+19+25+30+31)/7
Dog-directed aggression	Questionnaire items (23+24)/2
Excitability	Questionnaire items (62+63+64+65+66+67)/6
Dog rivalry	Questionnaire items (32+33+34+35)/4
Attachment- and attention-seeking	Questionnaire items (68+69+70+71+72+73)/6
Non-social fear and touch sensitivity	Questionnaire items (38+41+43+44+47+48+49+50+51)/9
Chasing	Questionnaire items (27+74+75+76)/4
Separation-related problems (Elimination)	Questionnaire items (88+89)/2
Energy	Questionnaire items (91+92+93)/3
Trainability	Questionnaire items (2+3)/2

C-BARQ=Canine Behavioral Assessment and Research Questionnaire

### Questionnaire

We used the Thai version of the C-BARQ, which was developed and validated from the modified Duffy and Serpell version [18] by Boonhoh *et al*. [16]. This version of the C-BARQ was chosen because the questionnaire was developed and validated, considering the language and cultural differences [19].

There are 12 subscales of canine behavior in the Thai C-BARQ, including:

1. Stranger-directed aggression: A dog exhibits aggression toward strangers who approach it, its owner, or other family members, either within or outside the house premise.
2. Unfamiliar dog- or human-directed fear: A dog exhibits fearful behavior when approached by unfamiliar dogs or strangers.
3. Owner-directed aggression: A dog exhibits aggressive behavior toward its owner or other household members when provoked, groomed, stared at, or approached when eating or playing.
4. Dog-directed aggression: A dog exhibits aggressive behavior when approached directly by unfamiliar dogs.
5. Excitability: a dog reacts strongly to exciting or provocative situations, for example, going for a walk, doorbell ringing, the owner arriving home, and car trips and is difficult to calm down.
6. Dog rivalry: A dog exhibits aggressive behavior toward other familiar dogs in the household.
7. Attachment- and attention-seeking: A dog exhibits affection toward and seeks attention from the owner or other household members and becomes agitated when the owner pays attention to another person, dog, or animal.
8. Non-social fear and touch sensitivity: A dog exhibits fearful behavior to sudden or loud noises, traffic, unfamiliar objects, nail clipping, grooming, bathing, and/or examination by a veterinarian.
9. Chasing: A dog chases cats, birds, squirrels, and/or other small animals when given the opportunity.
10. Separation-related problems (elimination): A dog eliminates in undesignated areas when left alone at night or during the daytime.
11. Energy: A dog is active, energetic, playful, puppyish, boisterous, and/or always on the go.
12. Trainability: A dog obeys simple commands immediately.

### Questionnaire scoring method

Each subscale was scored using a Likert-type five-point scale (0–4) that measured the frequency and severity of each behavior. The scores were as follows: 0, Never; 1, seldom; 2, sometimes; 3, usually; and 4, always.

Some questions might have been unanswerable by some respondents; in these cases, the respondents were instructed to select “Not observed/Not applicable” or “N/A,” which was then categorized as a missing value. If more than 25% in each given subscale was “Not observed/Not applicable” or “N/A,” the value for the subscale was treated as missing and excluded from the subscale analysis. The missing values are shown in column 3 of Table-3.

**Table-3:** Descriptive data of the Thai C-BARQ 12 subscales.

Thai C-BARQ subscales	N	Missing values	Mean	Median	SD	Min	Max	25%	75%
Stranger-directed aggression	1637	190	1.21	1.10	0.96	0.00	4.00	0.40	1.80
Unfamiliar dog/human-directed fear	1563	264	1.02	0.88	0.91	0.00	4.00	0.25	1.50
Owner-directed aggression	1769	58	0.48	0.29	0.63	0.00	3.86	0.00	0.71
Dog-directed aggression	1488	339	1.40	1.00	1.17	0.00	4.00	0.00	2.00
Excitability	1638	189	2.53	2.67	0.88	0.00	4.00	2.00	3.17
Dog rivalry	1149	678	1.18	1.00	1.06	0.00	4.00	0.25	2.00
Attachment- and attention-seeking	1810	17	2.35	2.33	0.88	0.00	4.00	1.83	3.00
Non-social fear and touch sensitivity	1759	68	1.11	1.11	0.59	0.00	3.11	0.67	1.56
Chasing	1759	68	2.09	2.00	1.22	0.00	4.00	1.00	3.00
Separation-related problems (Elimination)	1753	74	0.69	0.00	1.10	0.00	4.00	0.00	1.00
Energy	1784	43	1.34	1.33	0.71	0.00	2.67	0.67	2.00
Trainability	1782	45	2.27	2.50	1.13	0.00	4.00	1.50	3.00

SD=Standard deviation, Min=Minimum, Max=Maximum, 25%=25^th^ percentile, 75%=75^th^ percentile, C-BARQ=Canine Behavioral Assessment and Research Questionnaire

### Statistical analysis

Descriptive statistical data of the Thai version of the C-BARQ, including population size (*N*), mean, median, standard deviation, minimum, maximum, 25^th^ percentile, and 75^th^ percentile, were calculated using Jamovi version 2.3 (The Jamovi project, Sydney, Australia). Spearman’s correlation was used to analyze the relationship among subscales and between the subscales and sociodemographic variables (Table-1). Values of coefficients (positive or negative) >0.01 with p ≤ 0.05 were considered statistically significant correlations.

## Results

### Thai C-BARQ 12 subscale data

The most complete subscale was “attachment and attention-seeking,” which had only 17 missing values. In contrast, “dog rivalry” contained the highest number of missing values, with 678. Considering each subscale score, “excitability” was found to have the highest mean score of 2.53, while “owner-directed aggression” had the lowest mean score of 0.48. The median for “separation-related problems (elimination)” had the lowest score (0.00), and the median for “excitability” was the highest (2.67). The minimum score of all subscales was 0, while the maximum score (4.00) was recorded in nine out of 12 subscales (Table-3). The lowest value of the maximum scores was for “energy” (2.67). At the 25^th^ percentile, “owner-directed aggression,” “dog-directed aggression,” and “separation-related problems (elimination)” scored 0; at the 75^th^ percentile, the highest score was for “excitability” (3.17) (Table-3).

### Relationships among Thai C-BARQ subscales

Regarding the relationship among subscales, significant negative correlations were detected between “unfamiliar dog–/human-directed fear” and “energy” (r = −0.053; p = 0.035), and between “unfamiliar dog–/human-directed fear” and “trainability” (r = −0.062; p = 0.014). The subscale “attachment- and attention-seeking” was positively correlated with “excitability” (r = 0.056; p = 0.023), “non-social fear and touch sensitivity” (r = 0.052; p = 0.029), and “chasing” (r = 0.074; p = 0.002). A positive association was also observed between “owner-directed aggression” and “energy” (r = 0.052; p = 0.029) (Table-4).

**Table-4:** Spearman’s correlations between 12 subscales of Thai C-BARQ.

	i)	ii)	iii)	iv)	v)	vi)	vii)	viii)	ix)	x)	xi)	xii)
i) Stranger-directed aggression	—											
ii) Unfamiliar dog/human-directed fear	0.017	—										
iii) Owner-directed aggression	−0.034	−0.004	—									
iv) Excitability	−0.003	0.014	0.008	—								
v) Dog-directed aggression	0.010	0.025	−0.010	0.020	—							
vi) Dog rivalry	0.017	0.008	0.015	0.034	−0.035	—						
vii) Attachment- and attention-seeking	−0.018	0.004	−0.021	−0.056*	−0.033	0.045	—					
viii) Non-social fear and touch sensitivity	0.041	0.029	0.007	−0.023	−0.010	0.029	0.052*	—				
ix) Chasing	0.038	−0.025	0.017	0.028	0.033	0.029	0.074**	-0.040	—			
x) Separation-related problems (elimination)	0.031	−0.038	−0.002	−0.025	0.018	−0.011	0.009	0.005	0.010	—		
xi) Energy	−0.027	−0.053*	0.052*	0.012	−0.001	0.021	0.041	0.024	0.020	−0.003	—	
xii) Trainability	−0.008	−0.062*	−0.037	−0.001	−0.051	0.032	0.036	0.007	0.000	−0.016	0.019	—

* p<0.05,

** p<0.01, ***p<0.001 (2 tailed). C-BARQ=Canine Behavioral Assessment and Research Questionnaire

### Factors associated with Thai C-BARQ subscales

#### Stranger-directed aggression and unfamiliar dog–/human-directed fear

We observed significant associations between subscales and variables or factors in this study. A score for stranger-directed aggression was negatively correlated with dog size (p < 0.001) and the frequency of walks (p < 0.001), while it was positively correlated with the number of dogs in the household (p < 0.001) and diet type. Biologically Appropriate Raw Food (BARF), homemade, and conventional diets were associated with the highest to lowest stranger-directed aggression, respectively (p = 0.005). The unfamiliar dog–/human-directed fear score was positively correlated with the owner’s age (p = 0.037), dog’s age (p = 0.044), and number of meals per day (p = 0.019); however, it was negatively associated with body size (p < 0.001) and dog’s gender (p = 0.006). Receiving no training (p = 0.028) and consuming BARF (p = 0.044) were associated with high levels of fearfulness toward unfamiliar dogs and humans (Table-5).

**Table-5:** Spearman’s correlations between Thai C-BARQ subscales and factors.

Thai C-BARQ subscales	Owner gender	Owner age	Home type	Dog size	Dog age	Dog gender	Dog reproductive status	Number of dogs in the household	Other species in the household	Training	Walking the dog/week	Feeding types	Meals/day	Left alone (h/day)
Stranger- directed aggression	−0.015	0.037	−0.048	−0.168***	0.028	0.018	0.041	0.128***	−0.028	0.029	−0.103***	0.070**	0.032	−0.010
Unfamiliar dog/human- directed fear	−0.004	0.053*	−0.021	−0.136***	0.051*	−0.070**	0.016	0.035	0.016	0.055*	0.024	0.051*	0.059*	0.022
Owner- directed aggression	0.029	−0.023	0	−0.166***	0.043	0.095***	−0.024	−0.050*	0.166***	0.076**	0.019	0.030	0.061*	0.010
Excitability	0.026	−0.042	0.086***	0.016	−0.080**	0.028	−0.105***	−0.030	−0.020	−0.009	0.012	−0.059*	0.023	0.02
Dog-directed aggression	0.029	0.057*	−0.068**	−0.041	0.104***	0.079**	0.065*	0.133***	−0.014	0.037	−0.048	0.070*	−0.035	0.015
Dog rivalry	−0.022	0.00	−0.095**	−0.067*	0.083**	0	0.039	0.263***	−0.018	0.062*	−0.108***	0.125***	−0.076**	0.028
Attachment- and attention- seeking	0.015	−0.049*	0.045	−0.044	−0.116***	−0.017	−0.128***	0.040	0.019	0.067**	−0.015	−0.010	0.096***	−0.016
Non-social fear and touch sensitivity	−0.034	−0.022	−0.034	−0.063**	0.103***	−0.071**	0.005	0.050*	−0.050*	0.078**	-0.072**	0.010	0.012	0.057*
Chasing	−0.031	0.017	−0.112***	0.103***	−0.031	−0.033	0.024	0.060*	−0.156***	0.021	-0.011	0.069**	−0.013	0.003
Separation- related problems (elimination)	0.070**	−0.069**	0.076**	−0.122***	−0.053*	0.044	−0.120***	−0.022	−0.034	0.015	0.005	−0.045	0.024	0.002
Energy	0.002	−0.097***	−0.026	0.097***	−0.337***	0.054*	−0.224***	−0.040	0.028	−0.028	0.066**	−0.035	0.071**	−0.013
Trainability	−0.001	0.046*	0.022	0.135***	−0.071**	0.001	0.016	−0.061*	−0.019	−0.163***	0.212***	−0.054*	−0.071**	−0.029

* p<0.05,

** p<0.01,

*** p<0.001 (2 tailed). C-BARQ=Canine Behavioral Assessment and Research Questionnaire

#### Owner-directed aggression and excitability

The owner-directed aggression score was positively associated with male dogs (p < 0.001), the presence of other species in the household (p < 0.001), no training (p = 0.001), and the number of meals per day (p = 0.01); however, it was negatively correlated with dog body size (p < 0.001) and the number of dogs in the household (p = 0.035). Excitability was observed to be dependent on several variables. Younger dogs (p = 0.001), neutered dogs (p < 0.001), dogs fed with BARF diet (p = 0.016), and those that lived in homes without yards (p < 0.001) (e.g., apartments and condominiums) were associated with a high excitability score (Table-5).

#### Dog-directed aggression and dog rivalry

Older dog owners (p = 0.028), older dogs (p < 0.001), male dogs (p = 0.002), neutered dogs (p = 0.013), multi-dog households (p < 0.001), feeding type (p = 0.007), and homes with yards (e.g., detached houses) (p = 0.008) were linked to an increased dog-directed aggression score. Similarly, some of these factors were found to be associated with dog rivalry scores (residential type, p = 0.001; older dogs, p = 0.005; greater number of dogs in the household, p < 0.001; and diet type, p < 0.001). In addition, smaller body size (p < 0.001), untrained dogs (p = 0.036), lower dog walking times per week (p < 0.001), and fewer daily meals (p = 0.01) were also factors associated with dog rivalry behavior levels (Table-5).

#### Attachment- and attention-seeking, and non-social fear and touch sensitivity behaviors

The attachment- and attention-seeking score was found to be correlated with younger dog owners (p = 0.036), younger dogs (p < 0.001), intact dogs (p < 0.001), untrained dogs (p = 0.004), and several daily meals (p < 0.001). The non-social fear and touch sensitivity score were related to smaller size dogs, older dog ages, female dogs, a greater number of dogs in the household, the absence of other animals in the household, untrained dogs, routines with less dog walking time, and longer periods of dogs being left alone in the home (Table-5).

#### Chasing and separation-related problems (elimination)

The chasing score correlated with more space in the home (p < 0.001), larger dog size (p < 0.001), a greater number of dogs in the home (p = 0.012), absence of other species in the home (p < 0.001), and diet type (p = 0.004). The separation-related problem (elimination) score was correlated with male dog owners (p = 0.003), younger dog owners (p = 0.004), homes with no yard (p = 0.001), smaller dog sizes (p < 0.001), younger dogs (p = 0.026), and intact dogs (p < 0.001) (Table-5).

#### Energy and trainability

The rating for energy was associated with younger dog owners (p < 0.001), larger dogs (p < 0.001), younger dogs (p < 0.001), male dogs (p = 0.022), and intact dogs (p < 0.001), greater dog walking time routines (p < 0.001), and a greater number of daily meals (p = 0.003). The trainability score was dependent on dog owner age (p = 0.05), with older owners having correspondingly higher scores. Trainability was also associated with larger dogs (p < 0.001), young dogs (p = 0.003), lower number of dogs in the household (p = 0.01), trained dogs (p < 0.001), dog walking routine times (p < 0.001), lower number of daily meals (p = 0.003), and conventional diet (p = 0.022) (Table-5).

## Discussion

In the previous studies, the most frequent dog behavior problem reported by owners were excitability [3]; separation-related behavior [20]; and aggression [7, 21], particularly human-directed aggression [21]. The present study revealed that based on the high scores in the Thai C-BARQ online questionnaire, “excitability,” “attachment and attention-seeking,” and “chasing” were the top three dog behavior problems in Thailand. Excitability had the highest mean score in the present study, which was associated with homes with no yard, young dogs, and intact dogs. Our results are similar to the findings of Kobelt *et al*. [3], in addition to having children and toys in the household and first-time dog owners. Attachment- and attention-seeking behavior was associated with younger owners and dogs, which may be the result of first-time dog ownership and age-influenced dog behavior. Chasing was correlated with homes with yards and larger dogs because of the activity of the bigger dogs and available home area.

### Gender associated with dog behaviors

A previous study showed that male dogs develop behavior problems more frequently than female dogs [7]. Intact dogs had notably more behavior problems – including excitability, attachment- and attention-seeking, inappropriate elimination, and energy – compared with those of neutered dogs. Neutered dogs posed lesser problems in terms of aggression than intact dogs, while they commonly caused other behavior problems, including obsessive behavior, attention-seeking behavior, and house soiling [7]. In addition, in this study, male dogs showed markedly higher scores for owner-directed aggression, dog-directed aggression, and high energy, but female dogs showed more fear of strangers and unfamiliar dogs.

We found that younger male dog owners who lived in homes with no yard (e.g., apartments or condominiums) were correlated with separation-related behavior. This finding may result from young male owners having fewer management skills and experience with owning pet dogs. A limitation of this study is its lack of data related to previous dog ownership experience. This problem is not only present among owners but is also found among intact, young, smaller dogs. Savalli *et al*. [12] reported that female dog owners had a higher risk of separation-related behavior among their dogs, which differed from the results of the present study.

### Breed and age associated with dog behavior

Large breeds and younger dogs were reported to be easier to train in our study. These findings are similar to those of Hart and Hart [22], who reported that dog breeds influenced dog training [22]. Border collies and retrievers scored high on trainability [23], which is influenced by behavioral phenotypes in their genes [24]. Pet lifespans have been increasing, and research on dog aging has been increasing [25, 26]. The previous studies have reported that dog behavior and personality change throughout their lifetimes [25, 26]. Activity-independence educed quickly from puppyhood into adolescence and gradually reduced during later age periods [25]. Senior dogs made more mistakes than young dogs in a memory test and displayed higher avoidance of objects associated with new subjects [26]. These factors could be the reason for the lower trainability score observed in older dogs in our study. In addition, older dogs had higher unfamiliar dog-/human-directed fear scores similar to the results of Kutsumi *et al*. [27], who found that puppies responded more positively toward strangers than adult dogs. This could be attributed to the degeneration of their sensory organs.

### Factors associated with aggression behaviors

Male and small breed dogs, having other species in the homeand fewer daily meals were associated with a high risk of owner-directed aggression in our study. Smaller-sized dogs and the high number of dogs in the home were associated with stranger-directed aggression, while dog-directed aggression problems were found to be associated with older neutered dogs, homes with no yard, and having many dogs in the home. Hsu and Sun [10] reported owner-directed aggression associated with male and older dogs, neutered dogs, and female owners, while dog- and stranger-directed aggression associated with intrinsic and environmental variables, including homes with yard space, rural areas [10], older dogs [11, 28], and mixed breed dogs [12]. These problems have many factors involved, including socialization deficits, hormones, and genetic and neurophysiological components [29].

### Types of food associated with dog behaviors

We found that the BARF diet was associated with high risks of dog-directed aggression, stranger-directed aggression, unfamiliar dog–/human-directed fear, dog-directed aggression, dog rivalry and chasing, while a conventional kibble diet was associated with excitability and trainability. Dietary nutrition, particularly protein, is reported to have the potential to manipulate dog behavior [30]. Dodman *et al*. [30] reported a decrease in aggressive behavior in dogs fed with low-to-medium protein diets, while those fed a high-protein diet showed marked aggression. The BARF diet is categorized as a protein-rich diet and therefore exerts similar effects. Moreover, feeding dogs with tryptophan supplements and a low-protein diet might help decrease aggression [13] as tryptophan is converted into the neurotransmitter serotonin in the brain. However, animals need protein for maintaining health, wound healing, immune functions, and enzyme activities. In addition, low-protein diets or protein restriction can result in impaired body functions and muscle breakdown, leading to weakness, low energy, and altered behavior among dogs.

Conventional diets were associated with high excitability and trainability scores in the present study. Kibble food may contain a higher proportion of carbohydrates compared to that of BARF diets. In addition, high-calorific diets may influence excitability behaviors. Thus, Kibble food can be used as a treat for positive reinforcement of trained behaviors, such as sitting or staying [31].

## Conclusion

The present study illustrated the significant variables correlated to common behaviors among pet dogs in Thailand for the first time. These findings provide insight into dog behavioral issues experienced by their owners. The most frequently reported dog behavior problems were “excitability,” “attachment- and attention-seeking,” and “chasing.” While the “trainability” subscale also had a high mean score, it was not a problematic behavior of the dogs but rather was an admired behavior. Finally, owner-directed aggression had the lowest mean score in this study. These results provide veterinary practitioners with insights into factors related to common behavioral problems in dogs. These findings also serve as an important informational basis for further studies to promote dog–human relationships and reduce dog abandonment in Thailand.

## Authors’ Contributions

TW, CB, PS, and WB: Designed the study, prepared the questionnaire, and revised the manuscript. NW: Commented and advised on the experiment and revised the manuscript. WB: Collected and analyzed the data and drafted, revised, and edited the manuscript. All authors have read, reviewed, and approved the final manuscript.
